# Acquired Infectious Defect in the Atrioventricular Septum: A Complication of Aortic Valve Endocarditis

**Published:** 2018-10

**Authors:** Ali Hosseinsabet, Reza Mohseni-Badalabadi, Khalil Forozannia

**Affiliations:** *Tehran Heart Center, Tehran University of Medical Sciences, Tehran, Iran.*

**Keywords:** *Heart septum*, *Aortic valve*, *Endocarditis*

A 52-year-old man was admitted to our hospital with fever, weight loss, and dyspnea (New York Heart Association functional class III). Physical examinations revealed systolic (grade III/VI) and diastolic murmurs at the left parasternal border. The first transthoracic and transesophageal echocardiographic examinations showed left ventricular enlargement and hypertrophy with a reduced systolic function (ejection fraction=40%), moderate mitral regurgitation, bicuspid aortic valve with severe stenosis and regurgitation, mild tricuspid regurgitation, and multiple mobile particles attached to the right atrial (RA) wall near the base of the septal tricuspid leaflet. Blood culture showed alpha-hemolytic Streptococcus growth. The patient was treated with antibiotics for 10 days. On the 10th day of admission, the first atrioventricular block appeared. The second transthoracic and transesophageal echocardiographic examination revealed destructive aortic valve endocarditis, accompanied by vegetation on the aortic valve; posterior wall aortic root abscess formation with extension to the aorto-mitral intervalvular fibrosa region and the base of the anterior mitral leaflet, which had ruptured to the left ventricular outflow tract (LVOT) (Figure 1); perforation of the atrioventricular septum, resulting in an LVOT-to-RA communication with a systolic shunt (Figure 2); and a large mobile particle on the RA wall, adjacent to the aortic root (Video 1). These findings were documented in the operating room. The mechanical prosthetic mitral and aortic valves were replaced, the infectious tissue was debrided, the infectious materials were removed from the RA, and the LVOT-to-RA connection was repaired. Pathological examinations of the materials removed from the RA showed inflammatory tissue and thrombosis. After surgery, the patient developed complete heart block, which was treated with dual pacemaker implantation. He was discharged from the hospital in good condition. An acquired defect in the atrioventricular septum due to infectious endocarditis is the most possible scenario in this case. In the evaluation of patients with infectious endocarditis, the presence of any endocarditis complications should be investigated.

**Figure 1:**
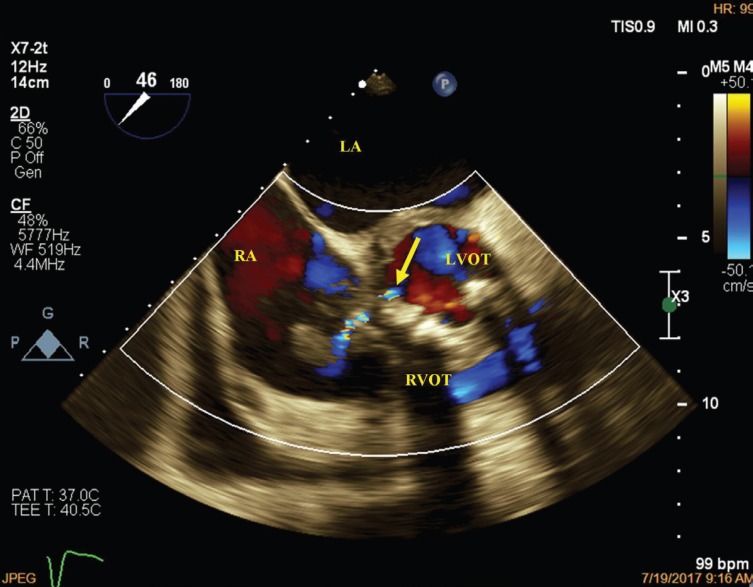
Transesophageal echocardiography in the 5-chamber view, demonstrating an aorto-mitral intervalvular fibrosa abscess rupturing to the LVOT (arrow).

**Figure 2:**
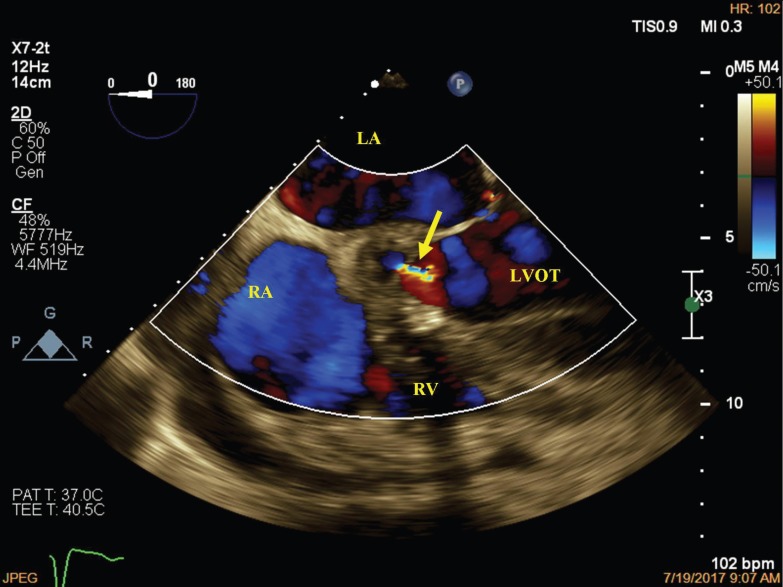
Transesophageal echocardiography in the modified short-axis of the aortic valve view, demonstrating an LVOT-to-RA connection with a systolic shunt (arrow).

***To watch the following videos, please refer to the relevant URLs:***

http://jthc.tums.ac.ir/index.php/jthc/article/view/886/808

Video 1. This video shows destructive aortic valve endocarditis, accompanied by vegetation on the aortic valve; posterior wall aortic root abscess formation with extension to the aorto-mitral intervalvular fibrosa region and the base of the anterior mitral leaflet, rupturing to the left ventricular outflow tract with an in-and-out flow; perforation of the atrioventricular wall, resulting in a left ventricular outflow tract-to-right atrium systolic shunt; and a large mobile particle on the right atrial wall adjacent to the aortic root.

## Notes:

***This paper should be cited as: ***Hosseinsabet A, Mohseni-Badalabadi R, Forozannia K. Acquired Infectious Defect in the Atrioventricular Septum: A Complication of Aortic Valve Endocarditis. J Teh Univ Heart Ctr 2018;13(4)193-194.

